# The Clinical Utilisation and Duration of Treatment with HER2-Directed Therapies in HER2-Positive Recurrent or Metastatic Salivary Gland Cancers

**DOI:** 10.3390/curroncol31090419

**Published:** 2024-09-21

**Authors:** Joseph Edward Haigh, Karan Patel, Sam Rack, Pablo Jiménez-Labaig, Guy Betts, Kevin Joseph Harrington, Robert Metcalf

**Affiliations:** 1 The Christie NHS Foundation Trust, Wilmslow Road Manchester, Manchester M20 4BX, UK; joseph.haigh3@nhs.net (J.E.H.);; 2 The Royal Marsden NHS Foundation Trust, Fulham Road, London SW3 6JJ, UK; 3 Department of Medicine, Universidad Autónoma de Barcelona, Plaça Cívica, Bellaterra, 08193 Barcelona, Spain; 4 The Institute of Cancer Research, Division of Radiotherapy and Imaging, 123 Old Brompton Road, London SW7 3RP, UK; 5 Manchester University NHS Foundation Trust, Oxford Road, Manchester M13 9WL, UK

**Keywords:** salivary gland cancer, HER2, trastuzumab, TDM1, ado-trastuzumab emtansine, trastuzumab deruxtecan

## Abstract

Salivary gland cancers (SGC) are rare tumours with limited availability of systemic therapies. Some SGC subtypes overexpress HER2, and this represents a potential therapeutic target, but the evidence base is limited. This study sought to analyse real-world data on the efficacy of HER2-directed therapies in SGC. This is a retrospective observational study using anonymised data from commercial compassionate-use access registrations and a privately funded pharmacy prescribing register. Treatment duration was defined as the time from drug initiation to treatment discontinuation. Kaplan–Meier analysis of treatment duration was performed using R for Windows (v4.3.2). A case report is also provided of an exceptional responder. Eighteen patients were identified who received HER2-directed therapies for HER2-positive recurrent/metastatic SGC, and complete data on treatment duration was available for 15/18. Histology was salivary duct carcinoma in 13/18 patients, adenocarcinoma NOS in 4/18, and carcinoma ex pleomorphic adenoma in 1/18. The median treatment duration was 8.3 months (95% CI: 6.41-not reached), and the range was 1.0–47.0 months. Choice of HER2-directed therapy varied, with ado-trastuzumab emtasine being the most common (9/18). At the time of analysis, HER2-directed therapy was ongoing for 9/15, discontinued due to disease progression for 4/15, discontinued due to toxicity for 1/15, and 1/15 was discontinued for an unspecified reason. An exceptional responder experienced a complete response with a treatment duration of 47.0 months. These real-world data are comparable to the median PFS observed with HER2-directed therapies in phase II trials and support the use of HER2-directed therapies in this group.

## 1. Introduction

Salivary gland cancers (SGC) are uncommon and diverse tumours that can be subclassified by anatomical site and histology. SGC represents 1–5% of head and neck cancers [1,2], and the global crude and age-adjusted incidences in 2022 were 0.70 and 0.56 per 100,000, respectively [3]. Sub-classification of SGC can also be on the basis of grade, with some types of SGC being more commonly low-grade, such as adenoid cystic carcinoma, and others being more frequently high-grade, such as salivary duct carcinoma [4].

Amplification of ERBB2 (Human Epidermal Growth Factor [HER2]) is most frequently described in typically high-grade SGC subtypes, including salivary duct carcinoma, adenocarcinoma not otherwise specified (NOS), and carcinoma ex pleomorphic adenoma, with a reported prevalence of 5–13% across all high-grade sub-types [4,5]. The prevalence of HER2-positivity, assigned by immunohistochemistry (IHC) and/or gene amplification by fluorescence in situ hybridisation (FISH), is reported in up to 13%, 39%, and 43% of adenocarcinoma NOS, carcinoma ex-pleomorphic adenoma, and salivary duct carcinoma, respectively [6].

Systemic treatment options are limited for SGC, and there is no clearly defined standard of care therapies for any SGC subtype in the recurrent and/or metastatic setting in the UK. However, generally accepted therapies for high-grade SGC may include cytotoxic chemotherapy and therapies targeting tumour molecular alterations like androgen-receptor blockers and HER2-directed therapies [1,2]. The latter, including ado-trastuzumab emtansine (TDM1) [7], trastuzumab [8], and pertuzumab [9], have shown significant efficacy in non-curable HER2-positive SGC. Consequently, The European Society for Medical Oncology (ESMO) and The American Society of Clinical Oncology (ASCO) guidance recommend consideration of HER2-directed treatment in recurrent/metastatic HER2-positive SGC [1,2]. However, HER2-directed therapies do not have regulatory approval for use in HER2-positive SGC in the UK, Europe, or the US, and there is no guidance on the selection of HER2-directed therapy or its sequencing alongside other anti-cancer therapies. 

In order to inform both clinical and healthcare system reimbursement decision-making in this context, there is a need to better understand the clinical use of HER2-directed therapies in SGC and the clinical outcomes, including the expected duration of therapy.

This study sought to describe the clinical utilisation of HER2-directed therapies in HER2-positive recurrent or metastatic SGC within the UK NHS and to determine the duration of therapy in the UK-wide, real-world setting, defined as data collected from clinical use within health care systems outside of clinical trials. 

## 2. Materials and Methods

This is a retrospective cohort study using commercial compassionate-use access registration data, privately funded treatment data, and a case report of a patient with an exceptional response to therapy.

To identify patients, Roche Products Ltd. provided anonymised data, on request, on all patients with HER2-positive recurrent or metastatic SGC who had accessed free-of-charge HER2-directed therapies within the UK NHS from 2017 to 2023, including details of the duration of therapy and prior therapies. Additional patients were identified through a pharmacy prescribing register at a single UK site.

Treatment duration was defined as the time from the date of treatment initiation to the date of treatment discontinuation. Kaplan–Meier analysis of treatment duration time was performed using Rstudio v2023.12.1+402 with the R programming language for Windows (v4.3.2) [10] and the ggsurvfit package [11]. Graphical analysis was performed using the ggplot2 package [12]. 

For a specific patient who received the greatest duration of HER2-directed therapy, the institutional records were reviewed to report the full case history.

The analysis was conducted in accordance with the UK Policy Framework for Health and Social Care Research. Case series review and data analyses were performed on anonymised data without any patient-level identifiable information. For the specific patient who had institutional clinical record review and is described as a case report, written informed consent was obtained under the Manchester Cancer Research Centre Biobank Research Tissue Bank Ethics (NHS NW Research Ethics Committee 8/NW/0092 [approved on 22 May 2018], superseded by 22/NW/0237 [approved on 30 August 2022]), and the patient provided informed written consent to the publication of de-identified clinical information and images.

## 3. Results

The primary aim of this study was to determine the choice of HER2-directed therapy and duration of treatment for patients with HER2-positive recurrent or metastatic salivary gland cancers treated with HER2-directed therapies. 

We identified 18 patients who received HER2-directed therapies between 2017 and 2023 (Table 1). Salivary duct carcinoma was the confirmed histology in 13/18 patients; one patient had a carcinoma ex pleomorphic adenoma, and the remaining four patients had a diagnosis of adenocarcinoma not otherwise specified (NOS).

All patients were confirmed to be HER2-positive. Two patients with HER2-positivity on tumour IHC testing underwent additional DNA next-generation sequencing (NGS) of their tumour sample. These showed ERBB2 copy number alterations, as well as TP53 alterations, in keeping with the typical genomic findings of salivary duct carcinoma [4]. Androgen receptor (AR) overexpression is frequently seen in salivary duct carcinoma. AR status was available for 15/18 patients; positive tumour staining for AR was seen in 12/15 cases, and all but one of these had received prior AR-directed treatment with bicalutamide with or without a luteinising hormone-releasing hormone (LHRH) agonist. Different LHRH agonists were used based on local availability, including triptorelin, leuprorelin, and goserelin. One patient received prior combination chemotherapy, and a further patient received combination chemotherapy between lines of HER2-directed therapies. The details of subsequent therapies were not available. 

**Figure 1:**
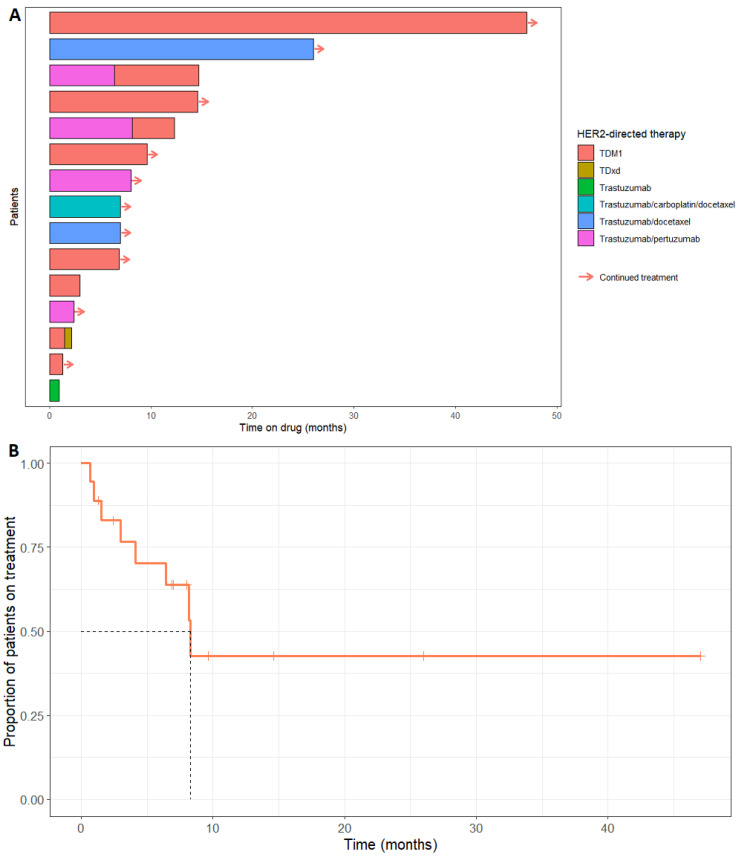
(**A**) Swimmers plot showing duration on HER2 directed therapy over time; (**B**) Kaplan–Meier analysis showing the proportion of patients continuing treatment with HER-2 directed treatment over time. The black dashed line shows the median duration on treatment. Censored patients for whom treatment was ongoing at time of analysis are marked with vertical orange lines.

The choice of HER2-directed therapy varied, with TDM1 being the most frequently adopted (Figure 1A). Nine patients received TDM1, five received trastuzumab and pertuzumab, six patients received trastuzumab with or without initiation chemotherapy, and one patient received trastuzumab deruxtecan (TDxd). Three patients received two lines of HER2-directed therapy (Table 1) due to disease progression after the first line. Complete data for time on HER2-directed therapy were available for 15/18 patients. The median time on HER2-directed therapy was 8.3 months (95% confidence interval: lower bound 6.41 months, upper bound not available), and the range was 1.0 to 47.0 months (Figure 1B). HER2-directed therapy was ongoing at the time of analysis for 9/15 patients. Four patients discontinued treatment because of disease progression (three of whom progressed through two lines of HER2-directed therapy), one patient stopped TDM1 due to treatment-related nausea and vomiting, and for one patient, the reason was not specified. 

**Figure 2:**
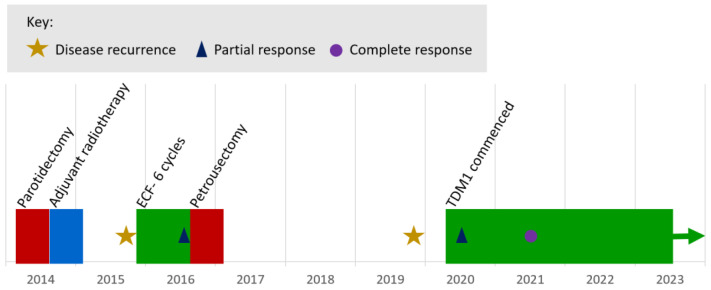
Treatment timeline of an index case report of durable complete response to ado-trastuzumab emtansine (TDM1) therapy for recurrent/metastatic HER2-positive salivary duct carcinoma. TDM1 therapy is ongoing at the time of analysis (green arrow).

The longest duration on treatment was 47.0 months, with treatment ongoing at the time of the analysis. This patient first presented 9 years prior (Figure 2), undergoing surgical resection and selective neck dissection for a pT1pN0 poorly differentiated salivary duct carcinoma of the right parotid gland with perineural invasion and close excision margins, followed by adjuvant radiotherapy. Within 12 months, there was a confirmed, inoperable, loco-regional recurrence involving the cavernous sinus. The patient received six cycles of epirubicin, cisplatin, and 5-fluorouracil with partial response, followed by a petrosectomy and resection of recurrent disease at the internal acoustic meatus. Three years later, there was a clinical and radiological progression of the disease. Immunohistochemical staining of the tumour recurrence was negative for AR expression but strongly HER2-positive (using the Ventana anti-HER2/neu (4B5) rabbit monoclonal primary antibody). Targeted NGS of the original primary tumour using the Foundation One assay (Foundation Medicine^®^) showed ERBB2 gene amplification (copy number: 11), as well as CCND3 amplification, and a TP53 mutation (M237I).

**Figure 3:**
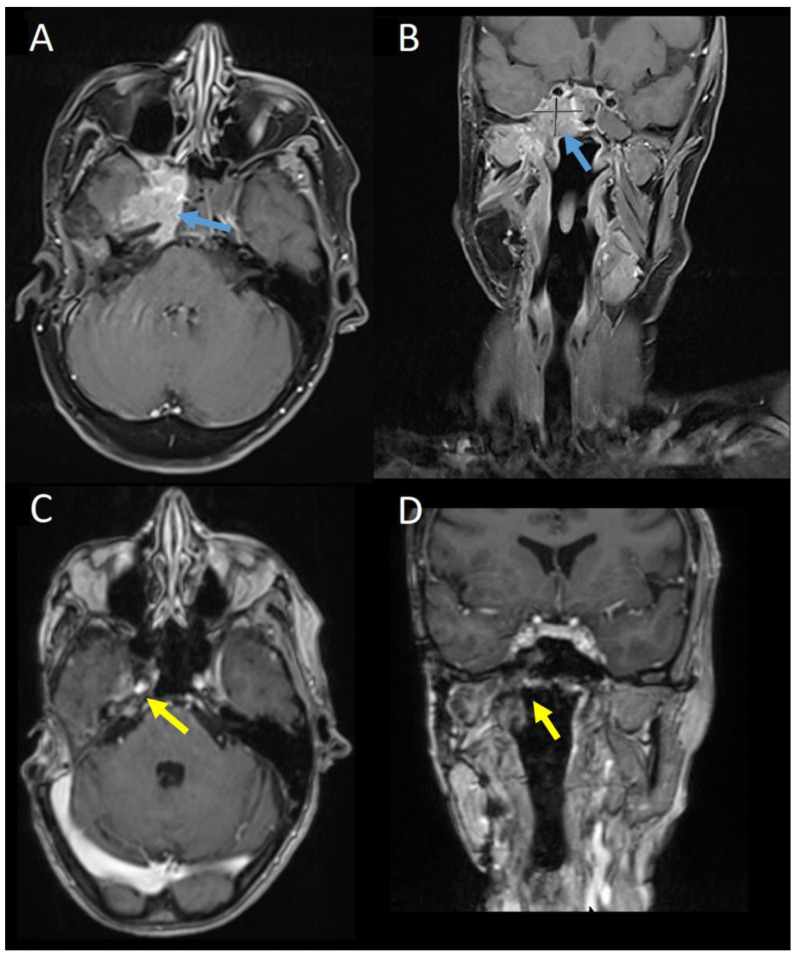
Gadolinium-enhanced MRI scan at baseline in (**A**) axial and (**B**) coronal planes and 38 months post-treatment in (**C**) axial and (**D**) coronal planes. The enhancing mass centred on the right cavernous sinus in (**A**,**B**) (blue arrows) is no longer apparent in (**C**,**D**) (yellow arrows).

Baseline gadolinium-enhanced MRI head prior to commencing TDM1 showed an unresectable tumour at the right cavernous sinus with involvement of the meninges, clivus, and petrous bone as well as the sphenoid sinus and masticator space (Figure 3A,B). CT with contrast did not demonstrate any distant metastases. Nuclear medicine cardiac ventriculography showed a left ventricular ejection fraction of 70%. Baseline symptoms were facial pain, paraesthesia of the right mandible, and unsteadiness. At this point, further surgery was not possible, and three-weekly TDM1, 3.6 mg/kg, was commenced.

At three months, there was evidence of clinical and radiological response. Gadolinium-enhanced MRI showed the mass centred on the cavernous sinus had reduced from 3.5 × 2.5 cm to 1.9 × 0.7 cm. The patient reported improvement in mobility and resolution of the facial pain. Serial imaging then showed further reduction in disease through to 15 months, from which no measurable disease was visible on MRI imaging. This remained the case on MRI imaging at 38 months post-treatment (Figure 3C,D). Six-monthly nuclear medicine cardiac ventriculography has shown a stable ejection fraction throughout treatment. Nine months after commencing treatment, the patient developed a stable, grade 1 increase in aspartate aminotransferase with no concurrent increase in bilirubin, which continues to be monitored and is considered to be drug-related. 

## 4. Discussion

In this report, we describe real-world employment of HER2-directed therapy for recurrent/metastatic SGC. TDM1 was the most frequently selected therapy. The median time on HER2-directed therapy was 8.3 months, and 13% of patients (2/15) exhibited durable responses to treatment. Overall, HER2-directed therapies were well tolerated, with only one patient reported as discontinuing due to treatment toxicity. In this case series, trastuzumab was mostly prescribed second line following AR-directed therapy in patients with AR overexpression. AR is strongly expressed in high-grade SGCs, and an ORR of 41.7% was observed in a phase II trial with leuprorelin and bicalutamide [13]. The ESMO guidelines acknowledge the uncertainty of the sequencing of AR-directed and HER2-directed therapies in AR-positive and HER2-positive SGC [2].

HER2-directed therapies, including trastuzumab, pertuzumab, tucatinib, TDM1, and TDxd, have been effectively used in multiple cancer types that over-express HER2. In breast, gastric, bladder, and non-small cell lung cancer, HER2 therapies are approved for use. In biliary tract and colorectal cancer, there are trial data to support the use of trastuzumab and pertuzumab, TDM1, and TDxd; however, further data are required to support their use in ovarian cancer [14].

The main evidence base for HER2-directed therapies in recurrent/metastatic SGC arises from the following phase II trials. Takahashi et al. conducted a single-arm study in 57 patients with HER2-positive salivary duct carcinoma given trastuzumab and docetaxel for 6 cycles followed by trastuzumab alone; 16% of patients had one previous line of treatment. The overall response rate was 70.2%, while median progression-free and overall survival were 8.9 months and 39.7 months, respectively. However, 60% of patients had grade 4 neutropaenia [8]. Li et al. observed an overall response rate of 90% in 10 patients with HER2-amplified SGCs receiving TDM1; five patients had a complete response. Previous trastuzumab use was not an exclusion. ERBB2-amplification by NGS is closely correlated with FISH and IHC [7]. In our case series, when targeted NGS was performed, this also showed ERBB2 copy number gain. Pertuzumab-trastuzumab demonstrated an overall response rate of 60% in 15 patients with HER2 amplified and/or overexpressed SGC. Median progression-free survival was 8.6 months, and median overall survival was 20.4 months. One patient with a HER2 gain-of-function mutation (HER2 S310F mutation) had a progression-free survival of 11 months [9]. Data illustrating the efficacy of trastuzumab monotherapy for the management of HER2-positive SGC are lacking. Haddad et al. noted a median time to progression of 4.2 months with trastuzumab alone in patients with SGC that was HER2 2+ and 3+ on IHC. Importantly, the trial did not use FISH to confirm HER2 amplification in HER2 2+ tumours, and no patients with salivary duct carcinoma participated. Additionally, the study stopped early due to difficulty recruiting patients with HER2-positive SGC [15]. Notably, there are no data directly comparing the efficacy of HER2-directed regimens in SGC.

The main limitation of this study is the small sample size. This is a common challenge when studying SGC due to the rarity of the disease. This is exacerbated by the heterogenous HER2-directed therapies employed. However, this is the largest reported dataset of its type, representing a national cohort of patients, and the outcomes presented here are similar to those reported by HER2-directed therapies in the trial setting discussed above.

Newer generation HER2-directed therapies with evidence of intracranial activity, such as TDxd and tucatinib, have been proven as effective options subsequent to TDM1 in HER2-positive breast malignancies [16]. Although it is currently unknown whether HER2-positive SGC exhibits a similar organotropism to the brain as HER2-positive breast cancer, examining the efficacy of these therapies in SGC is vital. In our case series, only a single patient received TDxd subsequent to TDM1. Further research into second-line HER2-directed therapies is warranted.

## 5. Conclusions

This report highlights the importance of screening for HER2-positivity in SGC, particularly for more aggressive subtypes like salivary duct carcinoma. Time on treatment seen in this series is similar to the median progression-free survival observed with trastuzumab–docetaxel and trastuzumab–pertuzumab in phase II trials.

## Figures and Tables

**Table 1:** Clinical characteristics of patients with recurrent/metastatic salivary gland cancer receiving HER2 targeted therapy for recurrent or metastatic HER2 positive salivary cancers.

Diagnosis	Androgen Receptor Status	HER-2 Status	Previous Systemic Treatment for Non-Curable Diseases	HER-2 Directed Therapy	Treatment Duration (Months)	Treatment Continued/ Discontinued	Reason for Treatment Discontinuation
Salivary duct carcinoma	Negative	HER-2 IHC: 3+ ERBB2 [HER-2] amplification)	Combination chemotherapy	Ado-trastuzumab emtansine	47.0	Continuing	Not applicable
Salivary duct carcinoma	Positive	HER-2 IHC: 3+ (ERBB2 [HER-2] amplification)	Androgen-directed therapy (NOS)	Ado-trastuzumab emtansine	9.7	Continuing	Not applicable
Salivary duct carcinoma	Positive	HER-2 IHC: 3+	Androgen-directed therapy (triptorelin and bicalutamide)	Ado-trastuzumab emtansine	6.9	Continuing	Not applicable
Salivary duct carcinoma	Negative	HER-2 IHC: 3+; FISH negative	None	Ado-trastuzumab emtansine	3	Discontinued	Disease progression
Carcinoma ex pleomorphic adenoma	Negative	Positive (IHC 3+)	None	Ado-trastuzumab emtansine	1.5	Discontinued	Disease progression
			TDM1, combination chemotherapy	Trastuzumab deruxtecan	0.7	Discontinued	Disease progression
Salivary gland carcinoma	Positive	Positive (test not specified)	Androgen-directed therapy (bicalutamide)	Ado-trastuzumab emtansine	1.3	Discontinued	Not specified
Salivary duct carcinoma	Positive	HER-2 amplification by FISH	Androgen-directed therapy (leuprorelin and bicalutamide)	Ado-trastuzumab emtansine	1	Discontinued	Treatment-related toxicity (nausea and vomiting)
Salivary gland carcinoma	Positive	Positive (test not specified)	Androgen-directed therapy (NOS)	Trastuzumab/ pertuzumab	6.4	Discontinued	Disease progression
			Androgen-directed therapy (NOS), trastuzumab/pertuzumab	Ado-trastuzumab emtansine	8.3	Discontinued	Disease progression
Salivary duct carcinoma	Not specified	HER-2 IHC: 3+	None	Trastuzumab/pertuzumab	8.2	Discontinued	Disease progression
			Trastuzumab/pertuzumab	Ado-trastuzumab emtansine	4.1	Discontinued	Disease progression
Salivary gland carcinoma	Not specified	Positive (test not specified)	Not specified	Trastuzumab/ pertuzumab	8.0	Not stated	Not specified
Salivary duct carcinoma	Positive	HER-2 IHC: 3+	Androgen-directed therapy (bicalutamide)	Trastuzumab/ pertuzumab	2.4	Continuing	Not applicable
Salivary duct carcinoma	Positive	HER-2 IHC: 2+; FISH negative	None	Docetaxel/ trastuzumab	26.0	Continuing	Not applicable
Salivary duct carcinoma	Positive	HER-2 IHC: 3+	Androgen-directed therapy (leuprorelin and bicalutamide)	Docetaxel/ trastuzumab	7.0	Continuing	Not applicable
Salivary duct carcinoma	Positive	HER-2 IHC: 3+	Androgen-directed therapy (NOS)	Trastuzumab	14.7	Continuing	Not applicable
Salivary duct carcinoma	Positive	HER-2 IHC: 3+	Androgen-directed therapy (leuprorelin and bicalutamide)	Carboplatin/docetaxel/trastuzumab	7.0	Continuing	Not applicable
Salivary duct carcinoma	Positive	Positive (test not specified)	Androgen-directed therapy (NOS)	Trastuzumab/ pertuzumab	Not stated	Not stated	Not specified
Salivary gland carcinoma	Not specified	HER-2 IHC: 3+	None	Trastuzumab	Not stated	Not stated	Not specified
Salivary duct carcinoma	Positive	HER-2 IHC: 3+	Androgen-directed therapy (goserelin and bicalutamide)	Trastuzumab	Not stated	Not stated	Not specified

## Data Availability

The data presented in this study are available on request from the corresponding author. The data are not publicly available due to the requirement to uphold the data sharing with relevant approved researchers as stipulated in the ethical approval.
